# CircFBXW7 in patients with T-cell ALL: depletion sustains MYC and NOTCH activation and leukemia cell viability

**DOI:** 10.1186/s40164-023-00374-6

**Published:** 2023-01-21

**Authors:** Alessia Buratin, Cristina Borin, Caterina Tretti Parenzan, Anna Dal Molin, Silvia Orsi, Andrea Binatti, Katharina Simon, Maddalena Paganin, Valentina Serafin, Enrico Gaffo, Geertruij te Kronnie, Pieter Van Vlierberghe, Silvia Bresolin, Stefania Bortoluzzi

**Affiliations:** 1 Department of Molecular Medicine, University of Padova, Padua, Italy; 2 Department of Biology, University of Padova, Padua, Italy; 3 Department of Maternal and Child Health, University of Padova, Padua, Italy; 4 Department of Surgical, Oncological and Gastroenterological Sciences, University of Padova, Padua, Italy; 5 Cancer Research Institute Ghent (CRIG), Ghent, Belgium; 6 Department of Biomolecular Medicine, Ghent University, Ghent, Belgium; 7 Onco-Hematology, Stem Cell Transplant and Gene Therapy Laboratory, IRP-Istituto Di Ricerca Pediatrica, Padua, Italy; 8 Interdepartmental Research Center for Innovative Biotechnologies (CRIBI), University of Padova, Padua, Italy

**Keywords:** CircFBXW7, T-cell Acute Lymphoblastic Leukemia, Circular RNA, Gene expression, Loss-of-function study

## Abstract

Circular RNAs (circRNAs) are emerging as new players in leukemogenic mechanisms. In patients with T-cell Acute Lymphoblastic Leukemia (T-ALL), the recent report of a remarkable dysregulation of circRNAs incited further functional investigation. Here we focus on circFBXW7, highly expressed in T-cells, with a notably high abundance of the circular compared to linear transcript of *FBXW7*. Two T-ALL patient cohorts profiled with RNA-seq were analyzed in comparison with five populations of developing thymocytes as normal counterpart, quantifying circRNA and gene expression. CircFBXW7 expression was very heterogeneous in T-ALL patients allowing their stratification in two groups with low and high expression of this circRNA, not correlated with *FBXW7* mutation status and T-ALL molecular subgroups. With a loss-of-function study in T-ALL in vitro*,* we demonstrate that circFBXW7 depletion increases leukemic cell viability and proliferation. Microarray profiling highlighted the effect of the circFBXW7 silencing on gene expression, with activation of pro-proliferative pathways, supporting a tumor suppressor role of circFBXW7 in T-ALL. Further, MYC and intracellular NOTCH1 protein levels, as well as expression of MYC target and NOTCH signaling genes were elevated after circFBXW7 depletion, suggesting an inhibitory role of circFBXW7 in these oncogenic axes. Plus, low circFBXW7 levels were associated with a particular gene expression profile in T-ALL patients, which was remarkably mirrored by the effects of circFBXW7 loss-of-function in vitro. CircFBXW7 depletion notably emerges as a new factor enhancing a proliferative phenotype and the activation of the MYC signaling pathway, key players in this aggressive malignancy.

**Supplementary Information:**

The online version contains supplementary material available at 10.1186/s40164-023-00374-6.

To the Editor,

CircRNAs are versatile regulators of cell biological activities and control oncogenic axes, with different mechanisms [1]. In pediatric T-cell Acute Lymphoblastic Leukemia (T-ALL), an aggressive hematologic malignancy for which there is urgent need to identify new disease mechanisms and therapeutic targets [2], we recently unearthed circRNA dysregulation and defined circRNA signatures of molecular genetic subgroups [3], but data about the role of circRNAs are limited [3, 4].

CircFBXW7, one of the most highly expressed circRNAs in T-cells [5], is downregulated in ALL of the B-cell lineage [5] and could play oncosuppressor functions in solid tumors [6] and in acute myeloid leukemia [7].

We quantified both circRNA and gene expression from RNA-seq data (Additional file 1) in two independent T-ALL pediatric patients’ cohorts (25 and 85 cases) and in sorted human thymocyte populations from healthy donors [3, 8]. CircFBXW7 expression, sustained and poorly variable in normal thymocytes, was instead heterogeneous, scattered over a wide interval, in T-ALL samples (Fig. 1A). T-ALL patients with low and high circFBXW7 expression were stratified using the median value, resulting lower than the normal counterpart average. T-ALL groups with low and high circFBXW7 expression were not significantly associated with *FBXW7* mutation state and T-ALL molecular subtypes with main driver genetic aberrancies (Additional file 1: Table S1, S2).

**Fig. 1:**
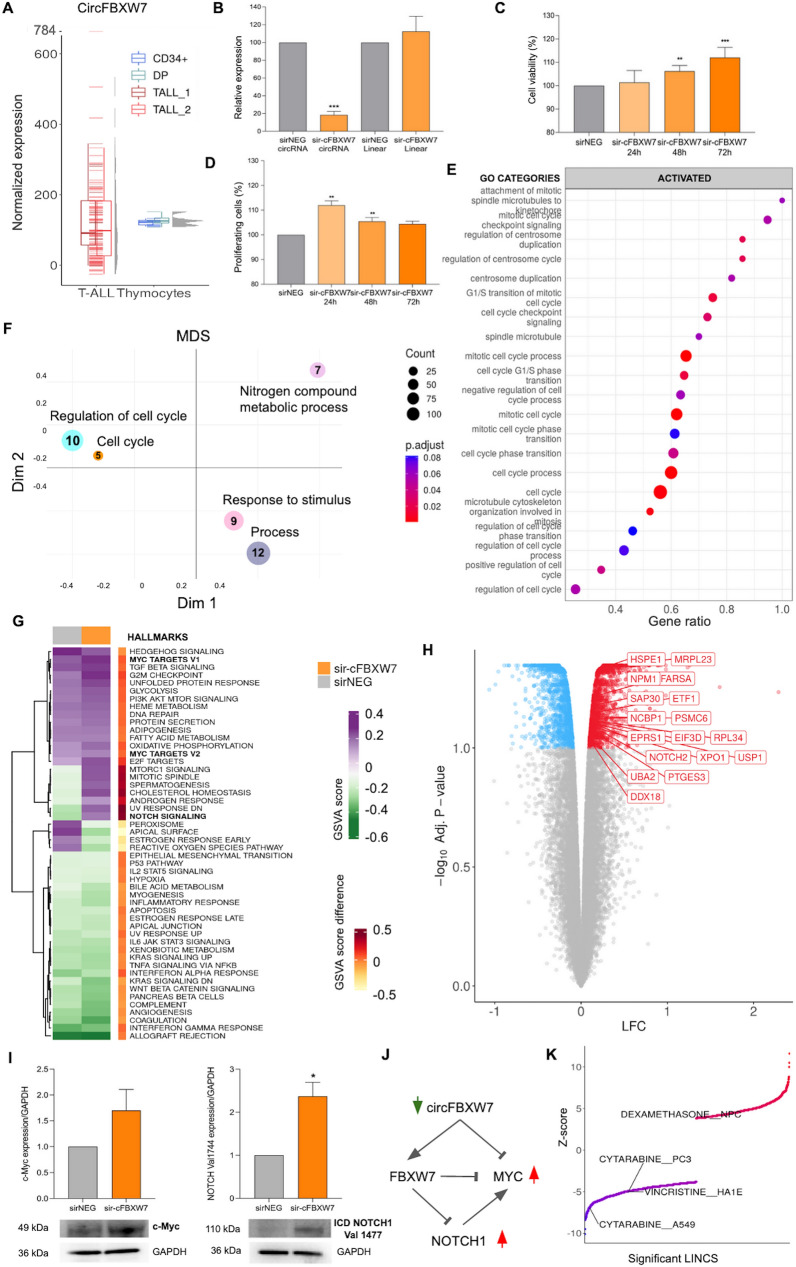
CircFBXW7 is depleted in T-ALL patients and its silencing has functional impact in T-ALL in vitro. **A** The boxplot shows the normalized expression of circFBXW7 in 110 patients with T-ALL of two cohorts (TALL_1 and TALL_2, 25 and 85 cases at diagnosis) and in the normal immature T-cell counterpart, represented by sorted human thymocyte populations CD34^+^ (CD34^+^CD4^−^CD1^−^, CD34^+^CD4^−^CD1^+^, CD34^+^CD4^+^) and DP (CD4^+^CD8^+^CD3^−^ and CD4^+^CD8^+^CD3^+^) from healthy donors; the gray density plots indicate the distribution of values across the ranges. **B** Real-time PCR quantification of circFBXW7 and FBXW7 mRNA in RPMI-8402 cell lines at 48 h after transfection in RPMI-8402 cells (Relative expression provided by ΔCt values using GAPDH as calibrator). **C** Cell viability upon circFBXW7 silencing, evaluated by MTT assay at 24, 48 and 72 h post transfection in RPMI-8402 cells. **D** Cell proliferation upon circFBXW7 silencing, evaluated by EdU assay at 24, 48 and 72 h post transfection in RPMI-8402 cells (mean ± SE from 3 independent experiments. **p < 0.01, ***p < 0.001). **E** Dot-plot of the activity of top GO terms significantly activated according to microarray gene expression profiling upon circFBXW7 silencing at 48 h, plotted in order of gene ratio. The size and the color of the dots represent respectively the number of genes associated with the significantly enriched GO term, and the adjusted p-value (BH) of the enrichment. **F** Multidimensional Scaling (MDS) plot based on Best-Match Average distance, representing the proximities of enriched GO term clusters. The dot size depends on the number of GO terms within each cluster. **G** Heatmap MSigDB Hallmark gene sets variation score and score difference in RPMI-8402 cells at 48 h after circFBXW7 silencing according to GSVA analysis. **H** Volcano plot of DEGs between high- and low-circFBXW7 expression groups in RPMI-8402. Labels show MYC targets and NOTCH signaling genes deregulated. **I** Western blot quantification of MYC and cleaved active intracellular NOTCH1 after circFBXW7 silencing and in control RPMI-8402 cells. **J** Schematic model of circFBXW7 relations with FBXW7, MYC, and NOTCH1 (red and green arrows indicate up and downregulation). **K** Connectivity score between the gene expression perturbation upon circFBXW7 silencing and drug LINCS signatures (positive and negative values are indicated in red and blue, respectively)

We set up a loss-of-function study in T-ALL in vitro, choosing RPMI-8402 and ALL-SIL with high baseline expression of circFBXW7 among other cell lines (Additional file 1: Table S3 and Fig. S1). When circFBXW7 was knocked down efficiently and specifically, with *FBXW7* mRNA unvaried (Fig. 1B), cell viability (MTT assay) significantly increased in both cell lines (Fig. 1C and Additional file 1: Fig. S2). Further studies in RPMI confirmed an increase of proliferating cell rate (EdU assay; Fig. 1D). Moreover, microarray expression profiling disclosed the regulatory networks perturbed by circFBXW7 depletion, with 2265 genes significantly differentially expressed (Additional file 2: Table S4). Chemokines and pro-apoptotic genes were downregulated (Additional file 1: Fig. S3). Cell-cycle, regulation of the transition between G1 and S phase, centrosome formation, and microtubule organization were among the most activated Gene Ontology (GO) functions (Additional file 3: Table S5; Fig. 1E), due to upregulation of pro-survival and pro-proliferative genes, positive regulators of mTOR signaling, oncogenic kinases and phosphatases. Cell-cycle regulation emerged also from dysregulated functions clustering (Fig. 1F). Two sets of MYC target genes (including NPM1, HSPE1, XPO1, USP1 and DDX18) and the NOTCH signaling pathway were among the MSigDB Hallmarks significantly upregulated upon circFBXW7 silencing (Fig. 1G, H). Western blot quantification indicated an increase in both MYC protein and NOTCH1 cleaved intracellular domain (Fig. 1I). The activation, upon circFBXW7 silencing, of MYC and NOTCH1 oncogenic axes in T-ALL could be compatible with evidence in breast cancer that circFBXW7 favors MYC degradation while stabilizing FBXW7 [6]. This effect, together with the well-known NOTCH1-dependendent MYC activation [9] suggest the hypothesis sketched in Fig. 1J of a feed forward loop converging on MYC.

The gene perturbation observed after circFBXW7 knock-down was significantly connected with gene expression signatures of FDA-approved drugs used for leukemia therapy (Fig. 1K). In line with a positive connectivity score with Dexamethasone effect, dose–response experiments showed that circFBXW7 depletion increases Dexamethasone sensitivity of T-ALL (Additional file 1: Fig. S4).

Further investigation of primary samples recognized that the effects of circFBXW7 loss-of-function in vitro remarkably mirrored the condition of the patient subset with reduced levels of this circRNA. Strikingly, the genes upregulated in T-ALL cases with low circFBXW7 expression were significantly enriched among those modulated upon its silencing in vitro, with pro-proliferative MsigDB Hallmarks overrepresented among the leading-edge genes commonly upregulated (Fig. 2A). Genes significantly differentially expressed upon silencing and with concordant expression variation in both patient cohorts (Fig. 2B) were enriched in biological functions and pathways linked to cell proliferation (Fig. 2C; Additional file 1: Fig. S5). *CDK1*, *CDC7* and *CDC25C*, the *YES1* oncogene, other genes associated with cell proliferation (*EML4, TTK, CDCA8, NUF2, NDC80,* and *CENP*) and stemness (*IKZF2*), and oncogenes targeted by MYC (*XPO1* and *USP1*) and NOTCH1 (*HES6*) were commonly upregulated. Tumor suppressors (*DUSP3*, *PRSS57* and *CDIP1)* were downregulated (Fig. 2B).

**Fig. 2:**
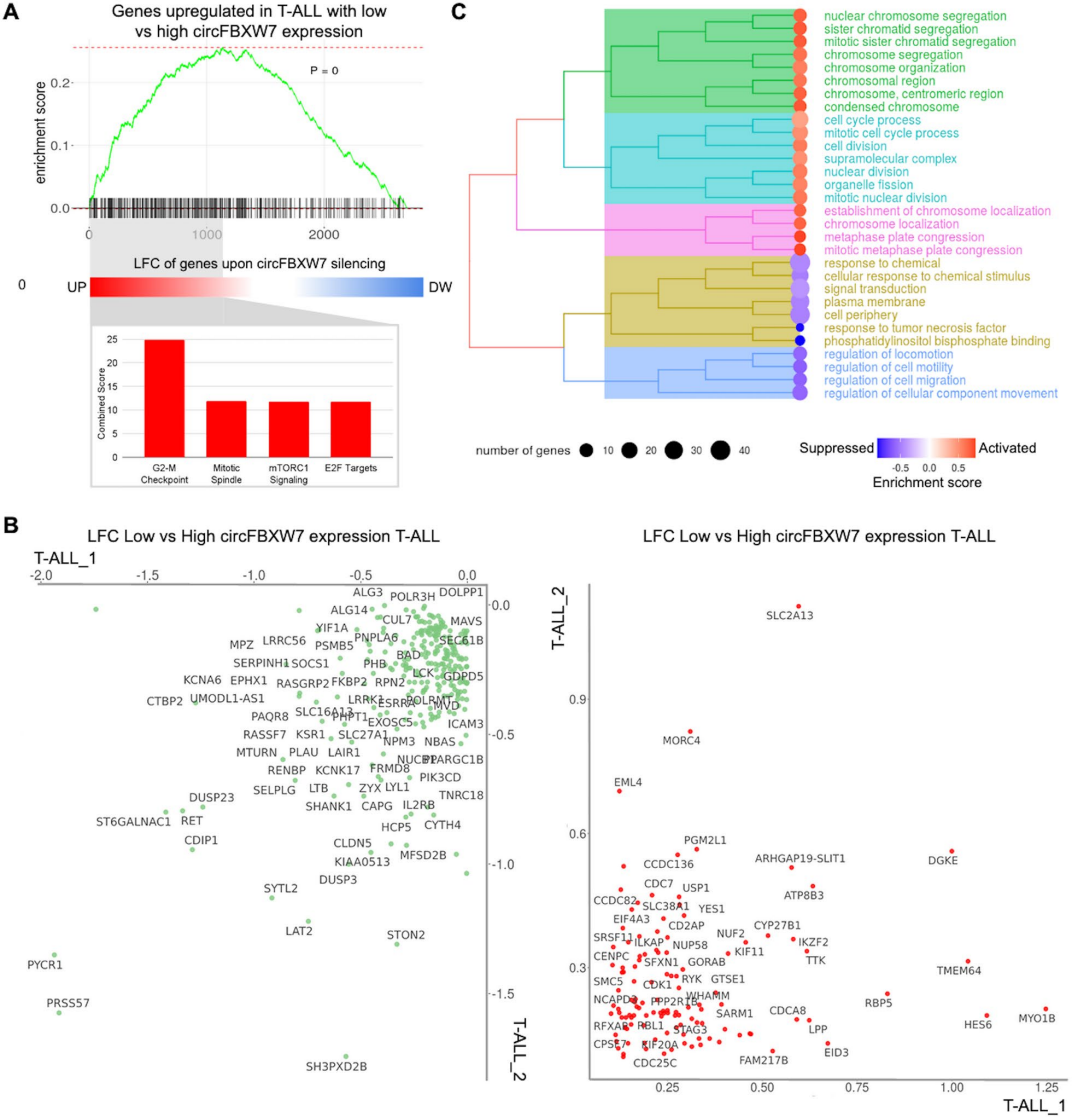
Characterization of T-ALL patients with a low circFBXW7 expression level. **A** Enrichment analysis of the custom signature defined by genes upregulated in T-ALL cases with low compared with cases with high circFBXW7 expression (Log Fold Change, LFC > 0.2) among the genes differentially expressed upon circFBXW7 silencing in RPMI-8402. The leading-edge subset of core genes driving the former custom enrichment was, in turn, enriched in specific MSigDB Hallmark gene sets, as shown in the bar plot in the bottom. **B** Scatterplots of LFC values of 274 genes significantly differentially expressed upon circFBXW7 silencing in the RPMI-8402 cell line and concordantly varied in both patient cohorts, comparing T-ALL cases with low against T-ALL with high circFBXW7 expression (up- and down-regulated genes are shown as red and green dots in separate panels) and **C** clustering of the functional categories most enriched among genes upregulated upon silencing in vitro and in T-ALL cases with low compared against T-ALL with high circFBXW7 expression

Our functional data of circFBXW7 silencing in T-ALL in vitro*,* concordantly with the recent report that its overexpression increases apoptosis in JURKAT cells [10]*,* strongly support the concept that circFBXW7 works as a tumor suppressor in this malignancy. Of novelty, functional and patient data concordantly indicated that the abnormal depletion of circFBXW7 in T-ALL is linked with a proliferative phenotype and represents a new factor that contributes to MYC and NOTCH1 hyperactivation key for leukemia onset and progression [11]. CircFBXW7 is an additional anti oncogenic product of the gene encoding the well-known tumor suppressor protein *FBXW7. FBXW7* inactivation due to somatic mutations in T-ALL resulted independent from circFBXW7 depletion, and these two molecules likely participate in an interconnected regulatory network.

CircRNAs are promising targets for innovative therapeutic strategies [12] and owing to its tumor suppressor role, circFBXW7 restoration or overexpression might be evaluated in the future to fight T-ALL.

## Supplementary Information

**Additional file 1. Table S1.** Variable distribution across T-ALL patients of the TALL_1 cohort with low- and high-circFBXW7 expression. **Table S2.** Variable distribution across T-ALL patients of the TALL_2 cohort with low- and high-circFBXW7 expression. **Table S3.** Primers used for qRT-PCR expression quantifications. **Figure S1.** Real-time PCR quantification of circFBXW7 expression level in four T-cell lines. **Figure S2.** Silencing of circFBXW7 in the ALL-SIL cell line. **Figure S3.** Dot-plot of the activity of top GO terms significantly suppressed upon circFBXW7 silencing at 48 hours, plotted in order of gene ratio. **Figure S4.** Drug sensitivity upon circFBXW7 silencing in T-ALL in vitro. **Figure S5.** Pathways enriched in genes modulated upon circFBXW7 depletion.

**Additional file 2. Table S4.** Differentially Expressed Genes upon circFBXW7 silencing in the RPMI-8402 cell line.

**Additional file 3. Table S5.** Gene Ontology (GO) terms significantly enriched upon circFBXW7 silencing in RPMI-8402 cells, accordign to Gene Set Enrichment Analysis (GSEA) of gene expression profiles.

## Data Availability

The RNA-seq data analyzed during the current study are freely available from the GEO repository GSE110636 [3] and can be obtained by any researcher through application at the NCBI dbGaP database [8].

## Abbreviations

CircRNA: Circular RNA
DEGs: Differentially expressed genes
GEP: Gene expression profile
GSEA: Gene set enrichment analysis
GSVA: Gene set variation analysis
LINCS: Library of integrated cellular signatures
MsigDB: Molecular signatures database
T-ALL: T-cell acute lymphoblastic leukemia
