# Supplementary material for: CircFBXW7 in patients with T-cell ALL: depletion sustains MYC and NOTCH activation and leukemia cell viability
Source: Exp Hematol Oncol. 2023 Jan 21;12:12. doi: 10.1186/s40164-023-00374-6 (PMC9863195; doi:10.1186/s40164-023-00374-6)
Supplement: Supplementary file 1 — Additional file 1. Table S1. Variable distribution across T-ALL patients of the TALL_1 cohort with low- and high-circFBXW7 expression. Table S2. Variable distribution across T-ALL patients of the TALL_2 cohort with low- and high-circFBXW7 expression. Table S3. Primers used for qRT-PCR expression quantifications. Figure S1. Real-time PCR quantification of circFBXW7 expression level in four T-cell lines. Figure S2. Silencing of circFBXW7 in the ALL-SIL cell line. Figure S3. Dot-plot of the activity of top GO terms significantly suppressed upon circFBXW7 silencing at 48 hours, plotted in order of gene ratio. Figure S4. Drug sensitivity upon circFBXW7 silencing in T-ALL in vitro. Figure S5. Pathways enriched in genes modulated upon circFBXW7 depletion. [file 40164_2023_374_MOESM1_ESM.pdf]

CircFBXW7 in patients with T-cell ALL: depletion sustains MYC and NOTCH activation and leukemia cell viability

Alessia Buratin[1,2,$], Cristina Borin[1,3,$], Caterina Tretti Parenzan, Anna Dal Molin[1], Silvia Orsi[1,2], Andrea Binatti[1], Katharina Simon[3], Maddalena Paganin[1,7], Valentina Serafin[4,7], Enrico Gaffo[1], Geertruij te Kronnie[1], Pieter Van Vlierberghe[5,6], Silvia Bresolin[3,7,*], Stefania Bortoluzzi[1,8,*]

[1] Department of Molecular Medicine, University of Padova, Padova, Italy
[2] Department of Biology, University of Padova, Padova, Italy
[3] Department of Maternal and Child Health, University of Padova, Padova, Italy
[4] Department of Surgical, Oncological and Gastroenterological Sciences, University of Padova, Padova, Italy
[5] Cancer Research Institute Ghent (CRIG), Ghent, Belgium
[6] Department of Biomolecular Medicine, Ghent University, Ghent, Belgium
[7] Onco-Hematology, Stem Cell Transplant and Gene Therapy Laboratory, IRP-Istituto di Ricerca Pediatrica, Padova, Italy
[8] Interdepartmental Research Center for Innovative Biotechnologies (CRIBI), University of Padova, Padova, Italy
[$] Equally contributing authors
[*] Corresponding authors

DATA SUPPLEMENT

Additional file 1 Methods 2

RNA-seq data analysis 2

Variant calling and impact prediction 2

Cell culture and transfection 3

RNA extraction and qRT-PCR 3

MTT and EdU assays 3

Cell viability versus drug dosage in circFBXW7 silenced cells 4

Gene expression profiling upon circFBXW7 silencing 5

Enrichment analysis 5

Additional file 1 Results 6

Supplemental Table 1. Variable distribution across T-ALL patients of the TALL_1 cohort with low- and high-circFBXW7 expression. 6

Supplemental Table 2. Variable distribution across T-ALL patients of the TALL_2 cohort with low- and high-circFBXW7 expression. 6

Supplemental Table 3. Primers used for qRT-PCR expression quantifications. 7

Supplemental Figure 1. Real-time PCR quantification of circFBXW7 expression level in four T-cell lines. 8

Supplemental Figure 2. Silencing of circFBXW7 in the ALL-SIL cell line. 9

Supplemental Figure 3. Dot-plot of the activity of top GO terms significantly suppressed upon circFBXW7 silencing at 48 hours, plotted in order of gene ratio. 10

Supplemental Figure 4. Drug sensitivity upon circFBXW7 silencing in T-ALL *in vitro*. 11

Supplemental Figure 5. Pathways enriched in genes modulated upon circFBXW7 depletion. 12

References 13

Supplemental Methods

RNA-seq data analysis

Patient RNA-seq data. We used two RNA-seq datasets of T-ALL patients; the T-ALL_1 is available in the Gene Expression Omnibus database with identification number GSE110636[1]; the T-ALL_2 was obtained through official application at the NCBI dbGaP database (project request #30803)[2].

CircRNA and gene expression quantification from RNA-seq data. CirComPara2 v0.1.2.1 bioinformatics pipeline[3], with default parameters, was used to identify and quantify both circular and linear transcript expression from RNA-seq data. CircRNA expression was normalized with a weighted trimmed mean of the log expression ratios[4]. Gene expression profiles were normalized using the DESeq2 normalization method. Genes not detected in more than 80% of samples were removed to filter out weakly expressed genes.

Gene differential expression analysis. Differentially expressed genes (DEGs) were assessed by DESeq2 using Benjamini-Hochberg correction, with an adjusted p-value ≤ 0.1.

All statistical analyses were performed in R version 4.0.2 (R Foundation for Statistical Computing, Vienna, Austria) and Bioconductor[5].

Variant calling and impact prediction

Sequence reads were aligned to the human reference genome (hg38) by STAR v2.7[6] in *Two-Pass* mode to characterize splice junction sites. The alignments were optimized according to the gold standard procedure of GATK4 for variant calling from RNA-seq data[7]. Variant calling was performed with HaplotypeCaller[8] and Mutect2[7] to detect both germline and somatic variants. The variants were annotated by SnpEff[9] and SnpSift[10] by using data available in dbSNP (build 155), ClinVar, Cosmic (v95), and GnomAD (v2.1.1). Prioritized driver variants were extracted considering functional impact (*HIGH* and *MODERATE*) and population allele frequency lower than 5%.

Cell culture and transfection

RPMI-8402 and ALL-SIL cells were suspended in RPMI 1640 Medium (Gibco Roswell Park Memorial Institute, ThermoFisher), at which was added 10% (v/v) FBS (Fetal Bovine Serum), 1% (v/v) GLU (Glutamine) and 1% (v/v) Penicillin/Streptomycin. Cells were seeded

at a concentration of 5.0×10^5 and cultured in an incubator containing 5% CO_2 at 37°C. We selectively silenced circFBXW7 using 30 pmol of the small interfering RNA (sir-cFBXW7 and of the control sirNEG (siRNA-NC, siRNA-ciR-FBXW7-1, both from[11]) synthesized by Thermo Fisher Scientific, Waltham, Massachusetts, USA.

Transfection of the different cell lines was performed using the Amaxa Biosystems (Lonza Sales Ltd., Basel, Switzerland) and Mirus solution (Bio™ *Transduce*IT™ Transduction Reagent, Thermo Fisher Scientific) and cells were seeded at a concentration of 1 x 10^5 cells/ml.

RNA extraction and qRT-PCR

RNA was extracted from RPMI-8402, ALL-SIL, JURKAT, and KOPT-K1 cell lines by Trizol (Thermo Fisher), and the RNA concentration and purity were measured by the Nanodrop 2000™ (Thermo Fisher). Complement DNA (cDNA) synthesis and qRT-PCR analysis were performed by SuperScript III First-Strand Synthesis System and SYBR green (Thermo Fisher). GAPDH was used as an internal reference to examine the expression level of circFBXW7 and FBXW7 mRNA. The primers involved are listed in **Additional file 1 Table 1**. The circFBXW7 expression level in cell line samples was analyzed by the $2^{-\Delta Ct}$ method, and all experimental data were expressed as the mean ± standard error (SE) of at least three independent replicates. For circRNA expression measures after silencing, the $2^{-\Delta\Delta Ct}$ method was applied, considering sirNEG as the calibrator.

MTT and EdU assays

Cell viability and proliferation after transfection with sirNEG and sir-cFBXW7 were evaluated using MTT (3-(4,5-dimethylthiazol-2-yl)-2,5-diphenyl tetrazolium bromide) and EdU (5-ethynyl-2'-deoxyuridine) assay, respectively.

For the MTT assay, concentration of the seeded cells was in a 96-well plate, performing three technical replicates and incubated with 10µl MTT (Sigma-Aldrich, St. Louis, Missouri, USA) for 2 hours after 48 and 72 hours post transfection. The absorbance was then detected at 540 nm using Victor3 TM 1420 Multilabel Counter (PerkinElmer, Waltham, Massachusetts, USA).

For the EdU assay, RPMI-8402 cells were seeded at a concentration of 1 x 10^5 cells per ml in 24-well plates, 50mM EdU (BCK-Edu488, Baseclick, Munich, Germany) solution was added per well and incubated for 4 hours. Cells were then fixed with 3.7% formaldehyde and permeabilized with Triton 0.5% X-100. Following, cells were incubated at room temperature

for 30 minutes with a reaction mix prepared according to the manufacturer's instructions. Cells signal fluorescence was acquired by the Cytomics FC500 Flow Cytometer (Beckman Coulter, Life Sciences, Indianapolis, Indiana, USA), and the results were analyzed by ImageJ software (National Institutes of Health, Bethesda, Maryland, USA). Cell viability was expressed as the percentage of positive cells stained with Edu. At least three biological replicates were performed with independent experiments for each cell line for both MTT and Edu assays.

Cell viability versus drug dosage in circFBXW7 silenced cells

After 24 hours RPMI-8402 transfection with sirNEG and sir-cFBXW7, cells were seeded at concentration of one million cells per ml in 96-well plates and they were treated with increased concentrations of Dexamethasone, Cytarabine, Vincristine and L-asparaginase (Sigma-Aldrich), or with DMSO as a control. Cells were incubated for 72 hours and then cell viability was assessed by an MTT test. The absorbance was then detected at 540 nm using Victor3 TM 1420 Multilabel Counter (PerkinElmer). The results are expressed as the percentage of viable cells, normalized to the control. At least three biological replicates were performed with independent experiments.

Western Blot analysis

The proteomes of RPMI-8402 cells were isolated between 48 and 72h after transfection with the RIPA lysis buffer (Sigma-Aldrich), following manufacturer's instructions. The protein concentrations were determined with the BCA Protein Assay Kit. SDS-polyacrylamide gel electrophoresis was performed using 4-20% Criterion TGX Stain Free Protein Gel (Bio-Rad, Hercules, California, USA) and the proteins were then transferred to poly-vinylidene difluoride (PVDF) membrane (Merck-Millipore, Billerica, Massachusetts, USA). After blocking with I-block 2% (Invitrogen, Waltham, Massachusetts, USA), the membranes were incubated at 4°C overnight with the primary antibodies for MYC (GTX 103436, Genetex, Irvine, California, USA), cleaved NOTCH1-Val1744 (D3B8, CS 4147, Cell Signaling Technology, Danvers, Massachusetts, USA), and GAPDH (GTX 627408, Genetex). The membranes were washed with tris-buffered saline with tween-20 (TBST) and incubated with the secondary antibody for 1h. At last, the protein bands were detected using Invitrogen™ iBright™ FL1500 Imaging System.

Gene expression profiling upon circFBXW7 silencing

Gene expression profiling (GEP) has been obtained in RPMI-8402 at 48 h after transfection (sir-cFBXW7 and sirNEG, two replicates each) using the GeneChip Human Clariom S™ (Affymetrix, Santa Clara, California, USA). In vitro transcription, hybridization, and biotin labeling of sscDNA were performed according to the WT assay starting from 100 ng of total RNA. Microarray data (CEL files) were generated using the default Affymetrix microarray analysis parameters (Command Console Suite Software).

CEL files were normalized using the robust multiarray averaging expression measure of the "affy" R package. For all analyses, genes without an Entrez gene ID were removed. All transcripts that do not have intensities greater than the median threshold were filtered out. A total of 11 762 genes were mapped to its Entrez gene ID. Differential expression analysis has been obtained using the "limma" R package (p-value adj.<0.1)[12].

Enrichment analysis

Gene set enrichment analysis (GSEA) on Gene Ontology terms was performed using the R packages "clusterProfiler"[13], "enrichplot" and "ViSEAGO"[14]. Term clustering was based on Best-Match Average distance[15].

Gene set variation analysis (GSVA) was used to provide an estimate of pathway activity by transforming an input gene-by-sample expression data matrix into a corresponding gene-set-by-sample expression data matrix. Hallmarks gene sets were taken from the Molecular Signatures Database (MSigDB)[16]. The GSVA score was calculated using the R package “GSVA”[17].

The connectivity between the differentially expressed genes upon circFBXW7 silencing and signatures of FDA-approved small molecule compounds (Library of Integrated Cellular Signatures, LINCS) has been calculated using QUADrATiC[18]. For each drug, a connection adjusted p-value estimation and a Z-Score have been obtained based on 5000 random signatures.

For all enrichment tests we used an adjusted p-value<0.1 as the cut-off value.

Additional file 1 Results

Additional file 1 Table 1. Variable distribution across T-ALL patients of the TALL_1 cohort with low- and high-circFBXW7 expression.

Association by Chi square test between the circFBXW7 domains in T-ALL patients and variables was accessed reporting the proportion of variable distribution across the two groups of patients, defined by circFBXW7 expression, and corresponding p-values.

Features	circFBXW7 high	circFBXW7 low	Total	p-value
Gender				0.144
Female	2 (15.4%)	5 (41.7%)	7 (28.0%)	
Male	11 (84.6%)	7 (58.3%)	18 (72.0%)	
Subgroup				0.916
HOXA	3 (23.1%)	2 (16.7%)	5 (20.0%)	
IMM	3 (23.1%)	2 (16.7%)	5 (20.0%)	
TAL-LMO	2 (15.4%)	3 (25.0%)	5 (20.0%)	
TLX1	2 (15.4%)	3 (25.0%)	5 (20.0%)	
TLX3	3 (23.1%)	2 (16.7%)	5 (20.0%)	
FBXW7 mutation				0.91
Absent	10 (76.9%)	9 (75.0%)	19 (76.0%)	
Present	3 (23.1%)	3 (25.0%)	6 (24.0%)	

Additional file 1 Table 2. Variable distribution across T-ALL patients of the TALL_2 cohort with low- and high-circFBXW7 expression.

Association by Chi square test between the circFBXW7 domains in T-ALL patients and variables was accessed reporting the proportion of variable distribution across the two groups of patients, defined by circFBXW7 expression, and corresponding p-values.

Features	circFBXW7 high	circFBXW7 low	Total	p-value
Gender				0.485
Female	11 (26.8%)	7 (20.0%)	18 (23.7%)	
Male	30 (73.2%)	28 (80.0%)	58 (76.3%)	
Subgroup				0.140
HOXA	4 (9.8%)	6 (17.1%)	10 (13.2%)	
LMO1/2	2 (4.9%)	2 (5.7%)	4 (5.3%)	
LMO2_LYL1	7 (17.1%)	2 (5.7%)	9 (11.8%)	
NKX2_1	1 (2.4%)	4 (11.4%)	5 (6.6%)	
TAL1	14 (34.1%)	8 (22.9%)	22 (28.9%)	
TAL2	0 (0.0%)	2 (5.7%)	2 (2.6%)	
TLX1	2 (4.9%)	5 (14.3%)	7 (9.2%)	
TLX3	11 (26.8%)	6 (17.1%)	17 (22.4%)	
FBXW7 mutation				0.609
Absent	27 (65.9%)	26 (60.5%)	53 (63.1%)	
Present	14 (34.1%)	17 (39.5%)	31 (36.9%)	

Additional file 1 Table 3. Primers used for qRT-PCR expression quantifications.

Gene	Primer forward	Primer reverse
GAPDH	3’-CCAGGGCATCCTGGGCTA-5’	5’-TTGAAGTCAGAGGAGACCACCTG-3’
circFBXW7	3’-TGACCCAGTAACTCCACTTCT-5’	5’-ACTAACAGTGTCACGAACTCCAG-3’
FBXW7	3’-GTGATAGAACCCCAGTTTCA-5’	5’-CTTCAGCCAAAATTCTCCAG-3’

Additional file 1 Figure 1. Real-time PCR quantification of circFBXW7 expression level in four T-cell lines.

Relative expression provided by ΔCt values using GAPDH as calibrator.

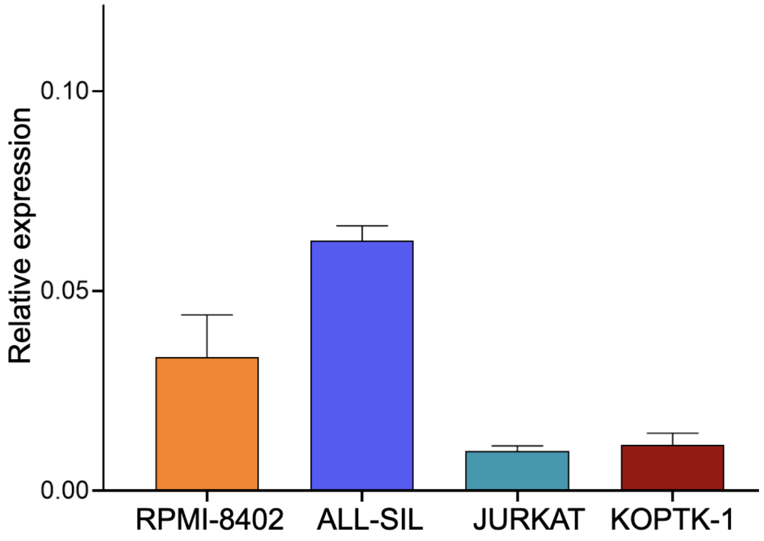

Additional file 1 Figure 2. Silencing of circFBXW7 in the ALL-SIL cell line.

A) Real-time PCR quantification of circFBXW7 and FBXW7 mRNA in ALL-SIL cell lines at 48 hours after transfection in ALL-SIL cells (Relative expression provided by ΔCt values using GAPDH as calibrator). **B)** Cell viability upon circFBXW7 silencing, evaluated by MTT assay at 24, 48 and 72 hours post transfection in ALL-SIL cells (mean ± SE from 3 independent experiments; $^{**}p < 0.01$).

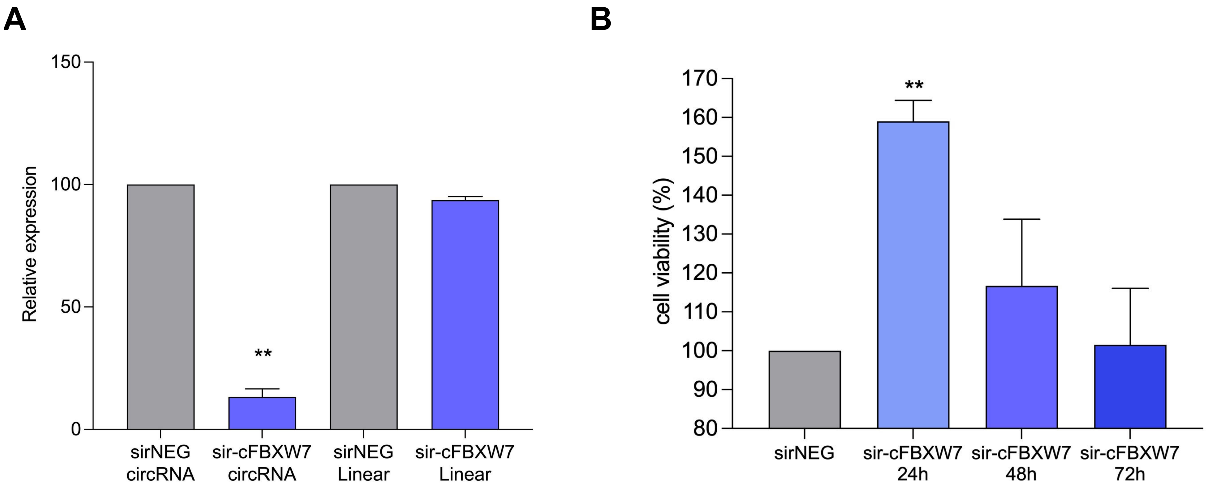

Additional file 1 Figure 3. Dot-plot of the activity of top GO terms significantly suppressed upon circFBXW7 silencing at 48 hours, plotted in order of gene ratio.

The size and the color of the dots represent respectively the number of genes associated with the significantly enriched GO term, and the adjusted p-value (BH) of the enrichment.

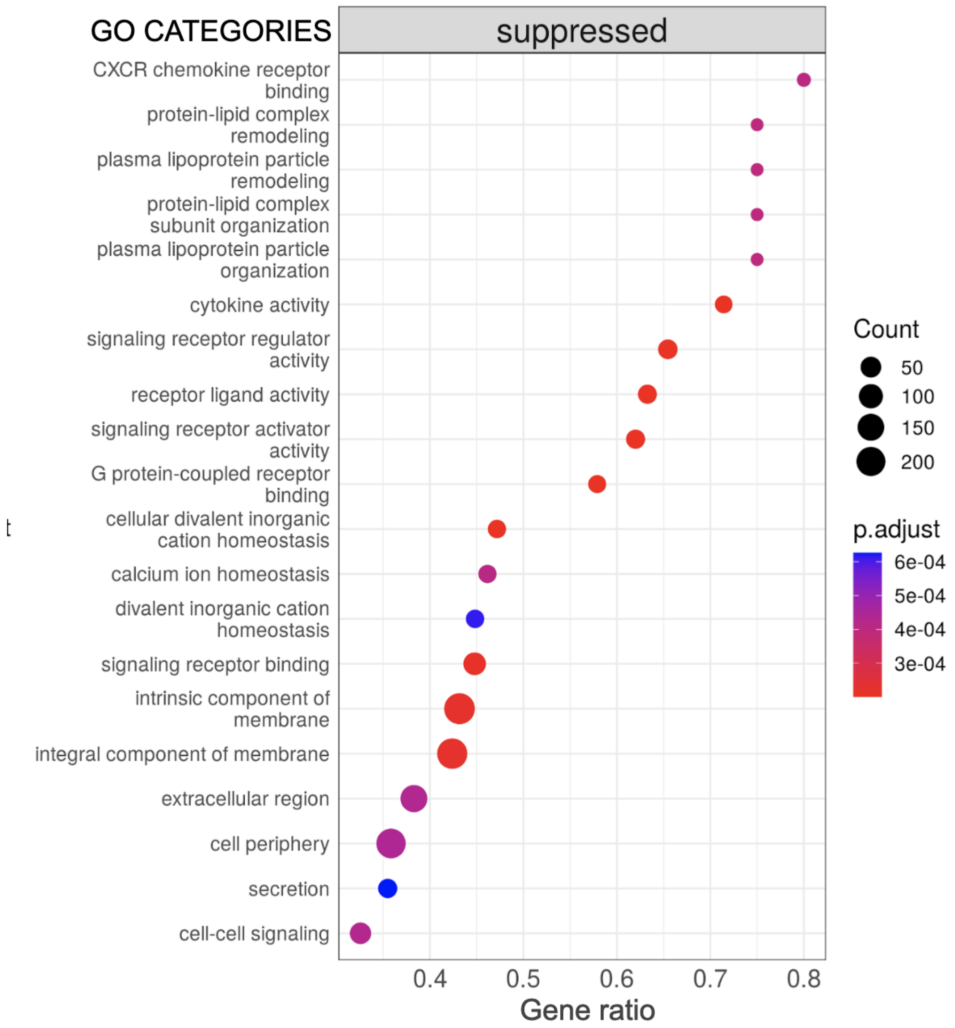

Additional file 1 Figure 4. Drug sensitivity upon circFBXW7 silencing in T-ALL in vitro. Cell viability was measured by MTT assay over drug dose upon circFBXW7 silencing in RPMI-8402 cell (DEX, Dexamethasone; L-ASP, L-asparaginase; ARAC, Cytarabine; VCR, Vincristine)(* p-value 0.034).

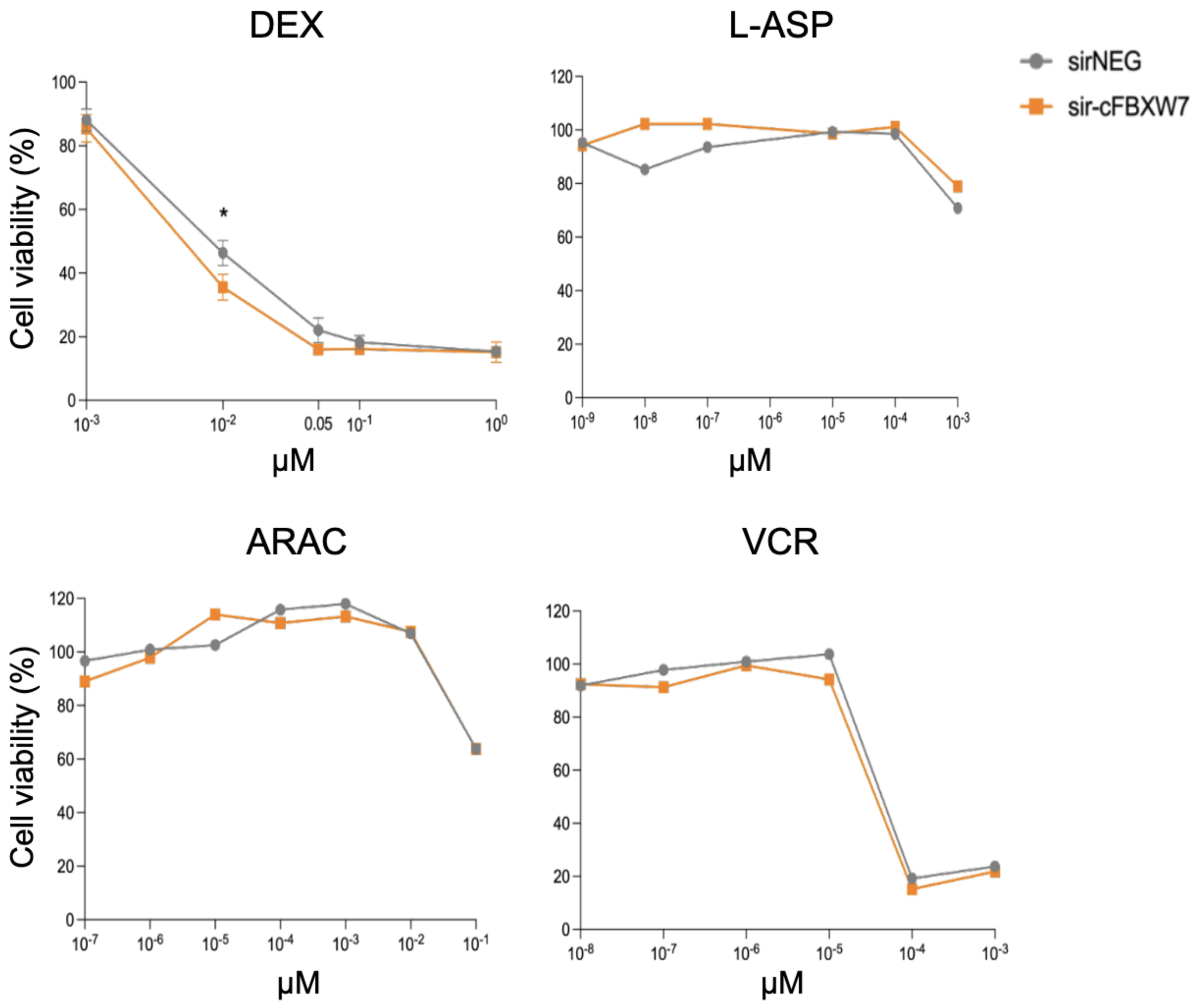

Additional file 1 Figure 5. Pathways enriched in genes modulated upon circFBXW7 depletion.

Enrichment score of Reactome and KEGG pathways considering genes significantly **(A)** upregulated and **(B)** downregulated upon circFBCXW7 silencing *in vitro* and with concordant expression variation in both cohorts of T-ALL patients.

A

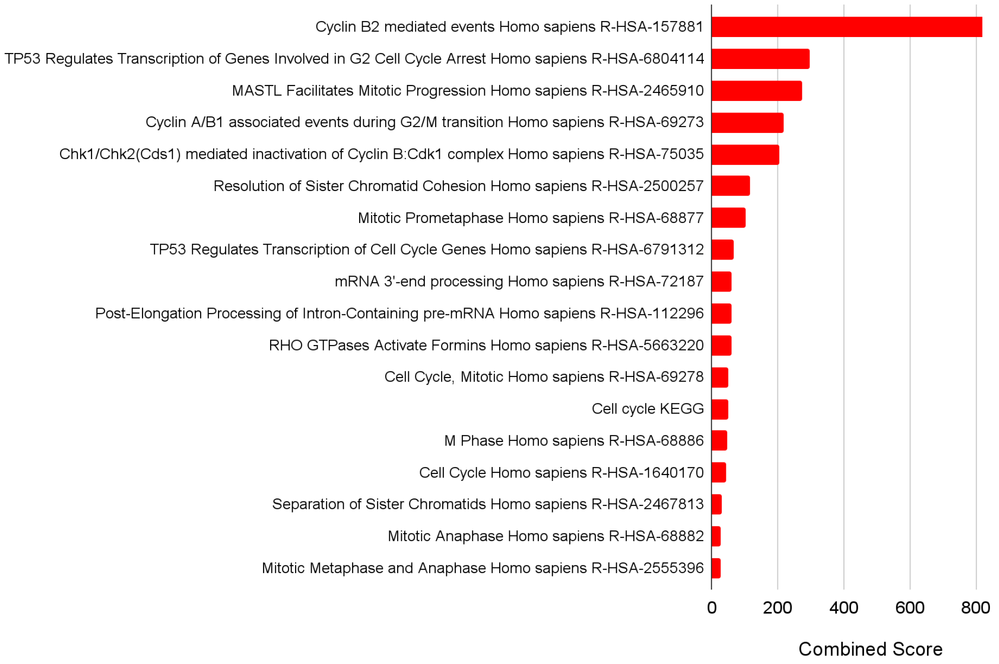

B

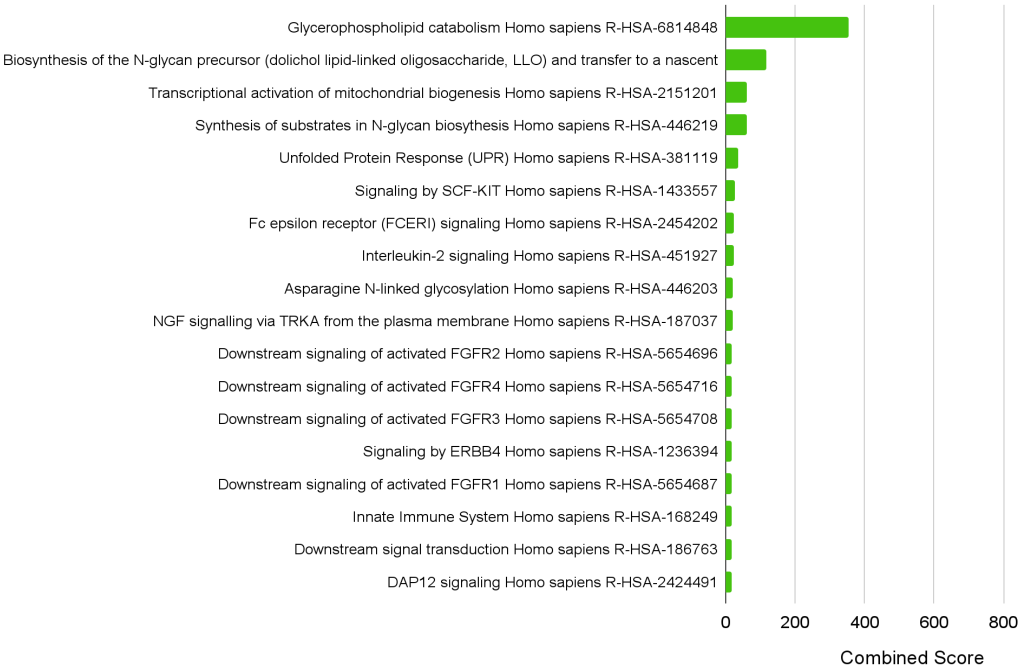

References

1. Verboom K, Van Loocke W, Volders P-J, Decaesteker B, Cobos FA, Bornschein S, et al. A comprehensive inventory of TLX1 controlled long non-coding RNAs in T-cell acute lymphoblastic leukemia through polyA+ and total RNA sequencing. Haematologica. 2018;103:e585–9.

2. Liu Y, Easton J, Shao Y, Maciaszek J, Wang Z, Wilkinson MR, et al. The genomic landscape of pediatric and young adult T-lineage acute lymphoblastic leukemia. Nat Genet. 2017;49:1211–8.

3. Gaffo E, Buratin A, Dal Molin A, Bortoluzzi S. Sensitive, reliable and robust circRNA detection from RNA-seq with CirComPara2. Brief Bioinform [Internet]. 2021; Available from: http://dx.doi.org/10.1093/bib/bbab418

4. Robinson MD, Oshlack A. A scaling normalization method for differential expression analysis of RNA-seq data [Internet]. Genome Biology. 2010. p. R25. Available from: http://dx.doi.org/10.1186/gb-2010-11-3-r25

5. Gentleman RC, Carey VJ, Bates DM, Bolstad B, Dettling M, Dudoit S, et al. Bioconductor: open software development for computational biology and bioinformatics. Genome Biol. 2004;5:R80.

6. Dobin A, Davis CA, Schlesinger F, Drenkow J, Zaleski C, Jha S, et al. STAR: ultrafast universal RNA-seq aligner. Bioinformatics. 2013;29:15–21.

7. Van der Auwera GA, O’Connor BD. Genomics in the Cloud: Using Docker, GATK, and WDL in Terra. O’Reilly Media; 2020.

8. Poplin R, Ruano-Rubio V, DePristo MA, Fennell TJ, Carneiro MO, Van der Auwera GA, et al. Scaling accurate genetic variant discovery to tens of thousands of samples [Internet]. Available from: http://dx.doi.org/10.1101/201178

9. Cingolani P, Platts A, Wang LL, Coon M, Nguyen T, Wang L, et al. A program for annotating and predicting the effects of single nucleotide polymorphisms, SnpEff: SNPs in the genome of Drosophila melanogaster strain w1118; iso-2; iso-3. Fly . 2012;6:80–92.

10. Cingolani P, Patel VM, Coon M, Nguyen T, Land SJ, Ruden DM, et al. Using Drosophila melanogaster as a Model for Genotoxic Chemical Mutational Studies with a New Program, SnpSift. Front Genet. 2012;3:35.

11. Yang Y, Gao X, Zhang M, Yan S, Sun C, Xiao F, et al. Novel Role of FBXW7 Circular RNA in Repressing Glioma Tumorigenesis. J Natl Cancer Inst [Internet]. 2018;110. Available from: http://dx.doi.org/10.1093/jnci/djx166

12. Smyth GK. Linear models and empirical bayes methods for assessing differential expression in microarray experiments. Stat Appl Genet Mol Biol. 2004;3:Article3.

13. Yu G, Wang L-G, Han Y, He Q-Y. clusterProfiler: an R Package for Comparing Biological Themes Among Gene Clusters [Internet]. OMICS: A Journal of Integrative Biology. 2012. p. 284–7. Available from: http://dx.doi.org/10.1089/omi.2011.0118

14. Brionne A, Juanchich A, Hennequet-Antier C. ViSEAGO: a Bioconductor package for

clustering biological functions using Gene Ontology and semantic similarity. BioData Min. 2019;12:16.

15. Pesquita C, Faria D, Bastos H, Ferreira AEN, Falcão AO, Couto FM. Metrics for GO based protein semantic similarity: a systematic evaluation. BMC Bioinformatics. 2008;9 Suppl 5:S4.

16. Liberzon A, Subramanian A, Pinchback R, Thorvaldsdottir H, Tamayo P, Mesirov JP. Molecular signatures database (MSigDB) 3.0 [Internet]. Bioinformatics. 2011. p. 1739–40. Available from: http://dx.doi.org/10.1093/bioinformatics/btr260

17. Hänzelmann S, Castelo R, Guinney J. GSVA: gene set variation analysis for microarray and RNA-seq data. BMC Bioinformatics. 2013;14:7.

18. O'Reilly PG, Wen Q, Bankhead P, Dunne PD, McArt DG, McPherson S, et al. QUADrATiC: scalable gene expression connectivity mapping for repurposing FDA-approved therapeutics. BMC Bioinformatics. 2016;17:198.
